# Carvedilol Prevents UV-Induced Immunosuppression and Skin Carcinogenesis through a Mechanism Independent of β-Blockade

**DOI:** 10.1016/j.xjidi.2025.100365

**Published:** 2025-03-24

**Authors:** Ayaz Shahid, Fanglong Dong, Bradley T. Andresen, Ying Huang

**Affiliations:** 1 Department of Biotechnology and Pharmaceutical Sciences, College of Pharmacy, Western University of Health Sciences, Pomona, California, USA; 2 College of Podiatric Medicine, Western University of Health Science, Pomona, California, USA

**Keywords:** β-Blocker, Carvedilol, Immunosuppression, Skin cancer, UVR

## Abstract

Exposure to UVR suppresses the immune system, which plays a primary role in skin cancer etiology. The β-blocker carvedilol prevents UV-induced skin cancer, but the mechanism is unknown. This study examined the effects of carvedilol and its enantiomers on UV-induced immunosuppression using contact hypersensitivity (CHS) response in SKH-1 mice. A single-dose UVR (224 mJ/cm^2^) strongly suppressed CHS, which was attenuated by intraperitoneal injection of carvedilol before UV exposure. Adoptive transfer of lymphocytes isolated from UV-irradiated mice to naïve mice without UV exposure triggered CHS suppression, which was not observed for lymphocytes isolated from carvedilol-treated mice. Topically applied carvedilol also prevented UV-induced CHS suppression. Both the β-blocking S-carvedilol and non–β-blocking R-carvedilol attenuated UV-induced CHS suppression. To evaluate the role of β2-adrenergic receptor, a knockout mouse model of β2-adrenergic receptor on the SKH-1 background was used. UV suppressed CHS in β2-adrenergic receptor–knockout mice, and carvedilol attenuated UV-induced CHS suppression in both genotypes. Furthermore, wild-type and knockout mice exposed to chronic UVR developed skin tumors with similar incidence, multiplicity, and tumor burden, whereas carvedilol inhibited skin tumor development in both genotypes. These data suggest that carvedilol prevents skin cancer not through β-blocking but through its activity overcoming UV-induced immunosuppression.

## Introduction

Skin cancer is the most commonly diagnosed cancer in the United States and the world (Ricotti et al, 2009). Despite the availability and promotion of sunscreen use for several decades, the incidence of skin cancer continues to rise (Hung et al, 2022; Planta, 2011), pointing to an urgent need for alternative preventive medicines. Exposure to the broad-spectrum UVR emitted from the sun or its individual components (UVA, UVB, and UVC) is the established risk factor for the development of most types of skin cancer owing to known effects of UV on the skin, including DNA damage, inflammation, and immunosuppression (González Maglio et al, 2016; Singh et al, 2018). In a UV-induced immunosuppressive microenvironment, skin cells that harbor UV-damaged DNA without repair may grow into tumors (Gupta and Mukhtar, 2002). It is only when DNA damage and immunosuppression coexist that it is possible for skin tumors to occur (Elmets et al, 2014). In both animal models and human participants, it has been demonstrated that UV irradiation causes the development immune tolerance so that the skin is unable to elicit sensitization to contact allergens (Bernard et al, 2019). Organ transplant recipients who receive long-term immunosuppressive treatment show a greater risk for skin cancer (Euvrard et al, 2003), further supporting the importance of immune system in skin carcinogenesis. Therefore, targeting UV-induced immunosuppression represents a potential strategy for protecting high-risk individuals against development of skin cancer.

Previous studies demonstrated that the β-blocker carvedilol prevents skin carcinogenesis in vitro and in vivo (Chang et al, 2015; Huang et al, 2017). Carvedilol is a receptor subtype nonselective β-blocker with additional α-blocking, nitric oxide–releasing, and antioxidant properties (Calò et al, 2005; Yue et al, 1992a, 1992b). In a large population-based cohort study, long-term carvedilol use is associated with reduced risk of several types of cancer (Lin et al, 2015). Mechanistic studies demonstrated that carvedilol attenuated UV-induced DNA damage (Chen et al, 2020a) and inhibited several oncogenic pathways involved in skin carcinogenesis, including the aryl hydrocarbon receptor (AhR) (Shahid et al, 2023), phosphoinositide 3-kinase/protein kinase B (Ma et al, 2019), MAPK/extracellular signal–regulated kinase (Cleveland et al, 2018), activator protein-1, cyclooxygenase-2, and NF-κB (Huang et al, 2017). However, the exact mechanism for carvedilol’s skin cancer preventive activity is still unknown. The main pharmacological function of a β-blocker is to block the binding and activation of β-adrenergic receptors by catecholamines (epinephrine and norepinephrine). Therefore, one possible mechanism for carvedilol’s cancer preventive activity is to antagonize the β-adrenergic receptors. Previous evidence has shown that activation of β-adrenergic receptors, particularly β2-adrenergic receptor (β2-AR), contributes to cancer development (Chida et al, 2008; Dhabhar et al, 2012; Hara et al, 2011; Saul et al, 2005; Sood et al, 2006; Spiegel et al, 2007; Yang et al, 2002; Yang and Eubank, 2013) and leads to reduced antitumor immunity (Qiao et al, 2019). β2-AR is expressed in most cell types in the skin (Gillbro et al, 2004) and is the primary subtype expressed on immune cells (Qiao et al, 2019). It has been shown that the immunosuppressive effects of catecholamines in tumor microenvironment are mainly mediated through the β2-AR (Mohammadpour et al, 2019; Qiao et al, 2019), and β2-AR is involved in the induction of regulatory T cells (Tregs) (Guereschi et al, 2013) and in the suppression of effector T cells (Bucsek et al, 2017). Most of the work for β2-AR used animal models that bear the already developed tumors. There are limited studies on the role of β2-AR in de novo carcinogenesis. It is unknown whether β2-AR is required for UV-induced immunosuppression and skin carcinogenesis and whether carvedilol’s skin cancer preventive activity is dependent on β2-AR blockade.

This study aimed to investigate the effects of carvedilol on UV-induced immunosuppression and whether this effect depends on the presence of β2-AR. To demonstrate UV-induced immunosuppression, a classical contact hypersensitivity (CHS) assay was conducted using SKH-1 hairless mice. Carvedilol was given either systemically or topically. The mechanism of carvedilol on the immune system was explored by adoptive transfer of mouse lymphocytes and splenocytes isolated from UV-exposed mice with or without carvedilol treatment. In addition, a mouse model of whole-body β2-AR knockout (KO) in the SKH-1 background and the wild-type (WT) SKH-1 littermate control mice were used in short-term and long-term UV exposure protocols to elucidate the role of β2-AR in UV-induced immunosuppression and skin cancer development. To our knowledge, this is previously unreported investigation on an unexpected activity for a Food and Drug Administration–approved cardiovascular drug (carvedilol) on immunosuppression.

## Results

### Systemically administered carvedilol prevented UV-induced immunosuppression

**Figure 1:**
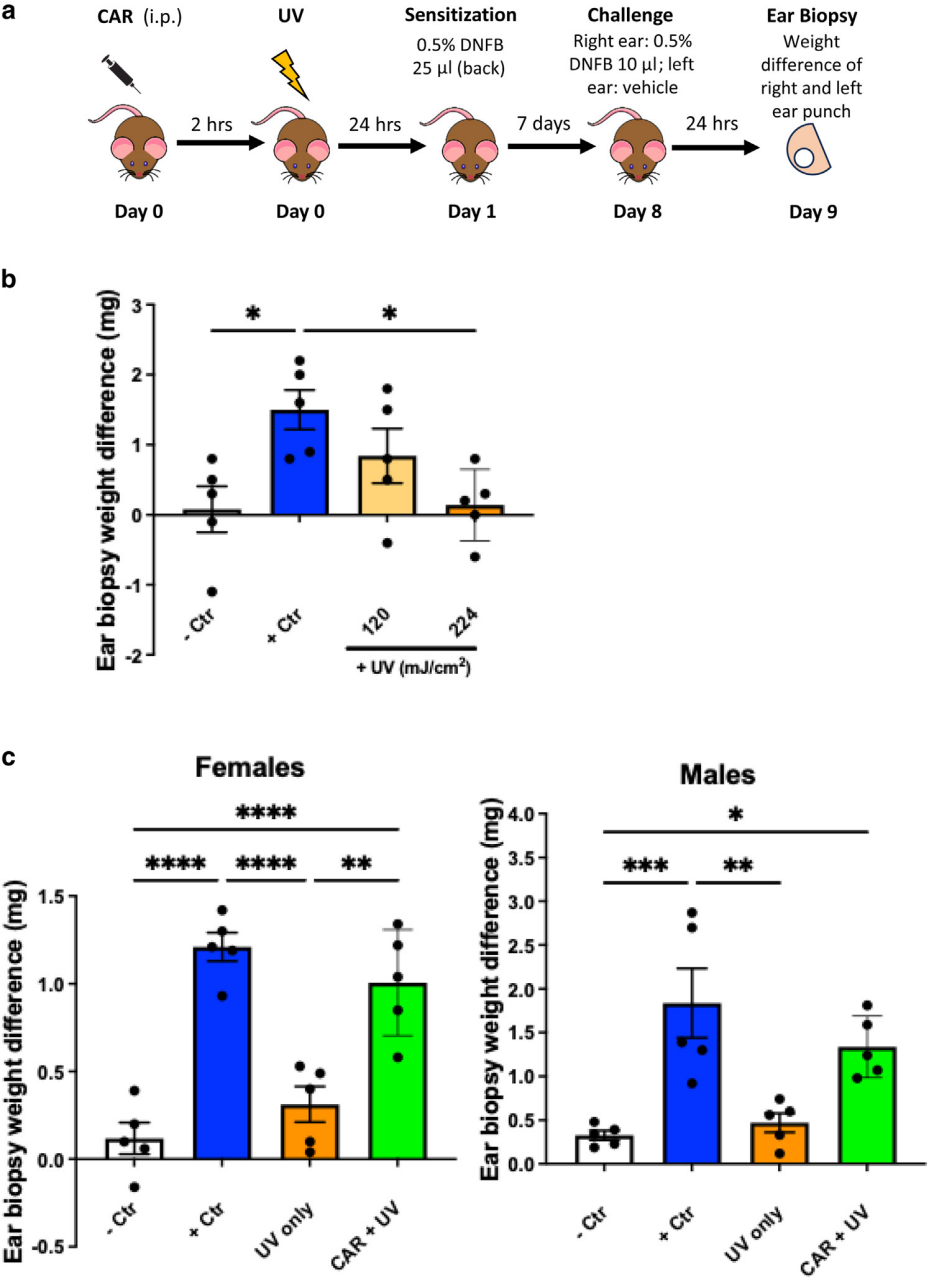
**Systemically administered CAR prevented UV-induced suppression of CHS reactions.** (**a**) Experimental scheme of CHS assay in the SKH-1 mice. Mice were treated with test agents (eg, CAR) through i.p. injection 2 h before UV exposure. Twenty-four h after, mice were sensitized with DNFB (25 μl) (0.5% in acetone:olive oil, 4:1, vol/vol) on UV-exposed back skin. After 7 days, the right ears were challenged with DNFB (10 μl, 0.5%), and the left ears were treated with vehicle. Twenty-four h after, 5-mm punch biopsies were obtained from both ears and weighed. (**b**) CHS model establishment in SKH-1 hairless mice and the effects of UV. The mice (male only) were irradiated with 2 different doses of UVR: 120 and 224 mJ/cm^2^. The data shown are the mean ± SEM of difference in ear biopsy weights (right ear minus left ear) (n = 5). (**c**) The mice (females and males) were pretreated with CAR (10 mg/kg) through i.p. injection 2 h before UVR (224 mJ/cm^2^). The data shown are the mean ± SEM of difference in ear biopsy weights (right ear minus left ear) (n = 5). One-way ANOVA with Tukey posthoc test was used to assess statistical differences. Multiplicity adjusted *P*-values for each comparison are shown: ∗*P* < .05, ∗∗*P* < .01, ∗∗∗*P* < .001, and ∗∗∗∗*P* < .0001. CAR, carvedilol; CHS, contact hypersensitivity; Ctr, control; DNFB, 2,4-dinitro-1-fluorobenzene; h, hour; i.p., intraperitoneal.

Immunosuppression induced by UV is commonly investigated using the CHS responses to low-molecular-weight contact allergens such as 2,4-dinitro-1-fluorobenzene (DNFB) (Schwarz et al, 2023). The CHS protocol is outlined in Figure 1a. To find the optimized UV doses that can induce immunosuppression in the SKH-1 mouse strain, the male mice were divided into the following 4 groups (Figure 1b): (i) non–UV-irradiated mice that received only the ear DNFB challenge on the right ear, serving as a negative control; (ii) non–UV-irradiated mice that received both sensitization and challenges with DNFB, serving as a positive control; (iii) mice that received 0.5-fold minimal-erythemal-dose UV exposure (120 mJ/cm^2^) before sensitization and challenges with DNFB; and (iv) mice that received 1× minimal-erythemal-dose UV exposure (224 mJ/cm^2^) before sensitization and challenges with DNFB. Compared with the negative controls, the positive control mice demonstrated a strong CHS reaction to the challenge of DNFB (*P* = .025) (Figure 1b). UVR dose-dependently reduced the CHS, with the higher dose significantly suppressing the CHS (*P* = .033), but the lower dose did not (*P* = .46) (Figure 1b). Therefore, the UV dose of 224 mJ/cm^2^ was selected for CHS experiments throughout this study.

A single-dose intraperitoneal injection of carvedilol (10 mg/kg) was given to the mice 2 hours before the UV exposure, followed by sensitization and challenges of DNFB (Figure 1a). This experiment was conducted in both female and male mice. In the female mice, UV exposure strongly suppressed the CHS (*P* < .0001). Carvedilol significantly reversed UV-induced CHS suppression (*P* = .001) (Figure 1c). The same protocol was applied to male mice, where UV also significantly suppressed the CHS (*P* = .003) but to a lesser degree than in the female mice. Carvedilol treatment showed the same trend in the male mice, which reversed the CHS suppression induced by UV but was not statistically significant (*P* = .060). These data are consistent with a previous report that UVB suppressed CHS equally in male and female mice (Reeve et al, 2012). The photoprotection effects have been shown with sex differences, with female mice showing favorable protection (Tongkao-On et al, 2021).

### Adoptive transfer experiment confirmed carvedilol’s preventive effects against UV-induced immunosuppression

**Figure 2:**
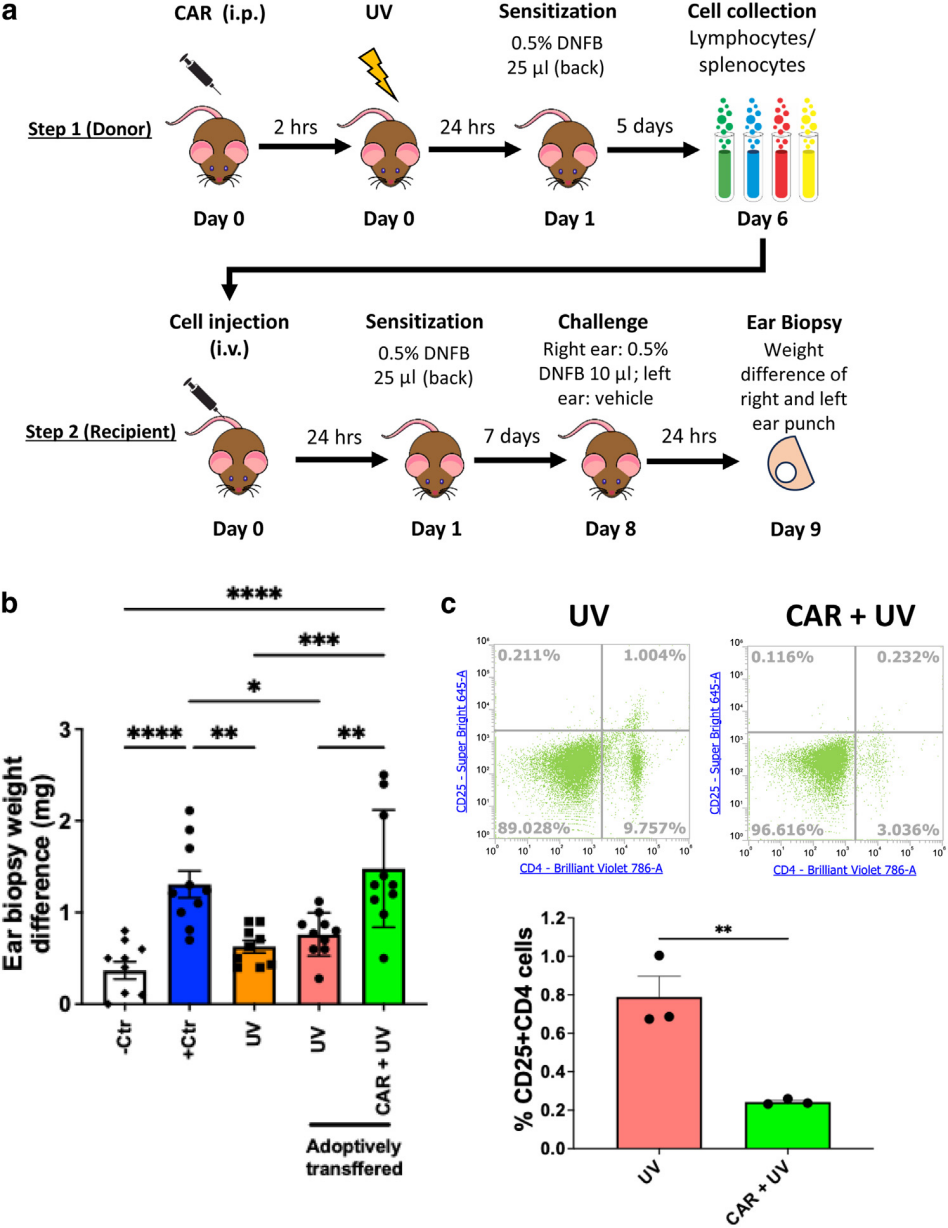
**Systemically administered CAR prevented UV-induced immunosuppression (adoptive transfer assay).** (**a**) Experimental scheme of the adoptive transfer assay in the SKH-1 mice. In step 1, on day 0, 2 groups of donor mice (male only) were pretreated with CAR (10 mg/kg, i.p.) or vehicle 2 hours before UV exposure (224 mJ/cm^2^) on the back (n = 5). Twenty-four hours after UV exposure, the mice were sensitized with DNFB on the back. Five days later (day 6), the mice were killed for collection of spleen and the inguinal, the axillary, and the brachial lymph nodes. In step 2, on day 0, lymph node cells and splenocytes obtained from the UV and CAR + UV groups of donor mice (n = 5) were pooled together per group and i.v. injected into the tail vein of naive mice (recipients) 24 hours before sensitization with DNFB. After 7 days, ears were challenged, and ear biopsy punch weight was measured after 24 hours. (**b**) CHS data obtained from the step 2 experiment. The mice (male only) were distributed into 5 groups: negative control without UV exposure but with DNFB treatment on the ear, positive control without UV exposure but with DNFB on the back and ear, UV-irradiated mice that received the same doses of DNFB on the back and ear, and recipient mice injected with lymph node cells and splenocytes that were obtained from the step 1 UV and CAR + UV donor groups (adoptively transferred), respectively. After 7 days, right ears were challenged, and ear swelling was measured by weight differences of right and left ear punch after 24 hours. Data are the mean ± SEM of difference in ear biopsy weights (n = 9∼10). (**c**) The single-cell suspensions of lymphocyte were obtained from UV and CAR + UV donor groups (n = 3), stained with antibodies and subjected to FACS analysis. One-way ANOVA with Tukey posthoc test was used to assess statistical differences. Multiplicity adjusted *P*-values for each comparison are shown: ∗*P* < .05, ∗∗*P* < .01, ∗∗∗*P* < .001, and ∗∗∗∗*P* < .0001. CAR, carvedilol; CHS, contact hypersensitivity; Ctr, control; DNFB, 2,4-dinitro-1-fluorobenzene; hr, hour; i.p., intraperitoneal; i.v., intravenously.

Because UV induces immunosuppression primarily through the activation and expansion of Tregs (Schwarz, 2005), we examined the effects of carvedilol on lymphocytes using an adoptive cell transfer assay. The experimental design is shown in Figure 2a. At step 1, cells in the skin-draining lymph nodes and splenocytes were isolated from vehicle-treated donor mice that were exposed to UV and sensitized with DNFB and from donor mice that were treated with 10 mg/kg carvedilol (intraperitoneally) 2 hours before UV exposure. At step 2, the pooled lymphocytes collected from 2 groups of donor mice (UV and CAR + UV) were intravenously injected into naive mice without UVR (recipient). After 24 hours, the recipients were sensitized with DNFB on the back, and 7 days later, the mice were challenged with DNFB in the right ear. The ear biopsy weight difference representing the ear swelling response showed that the recipients that were adoptively transferred with cells isolated from UV-irradiated donors were significantly reduced to 58% of the positive control group (*P* = .034) (Figure 2b), suggesting that immune cells with regulatory properties, possibly Tregs, developed in the UV-exposed donors. In contrast, CHS was not suppressed in animals injected with cells isolated from UV-exposed and carvedilol-treated mice (*P* = .003 for adoptively transferred UV vs CAR + UV groups), indicating that carvedilol may prevent UV-mediated induction of the suppressive immune cells (Figure 2b). To determine whether carvedilol prevents the induction of Tregs, splenocytes and lymph node cells were collected from mice that were exposed to UV (with or without carvedilol treatment) and sensitized with DNFB, and lymphocytes were collected 5 days later. The flow cytometry analysis indicated that carvedilol significantly reduced the percentage of CD4+CD25+ cells, which represent the Tregs (Figure 2c). The adoptive transfer and FACS experiments used male mice, confirming the treatment effects of carvedilol in the male mice.

### Topically administered carvedilol prevented UV-induced immunosuppression

**Figure 3:**
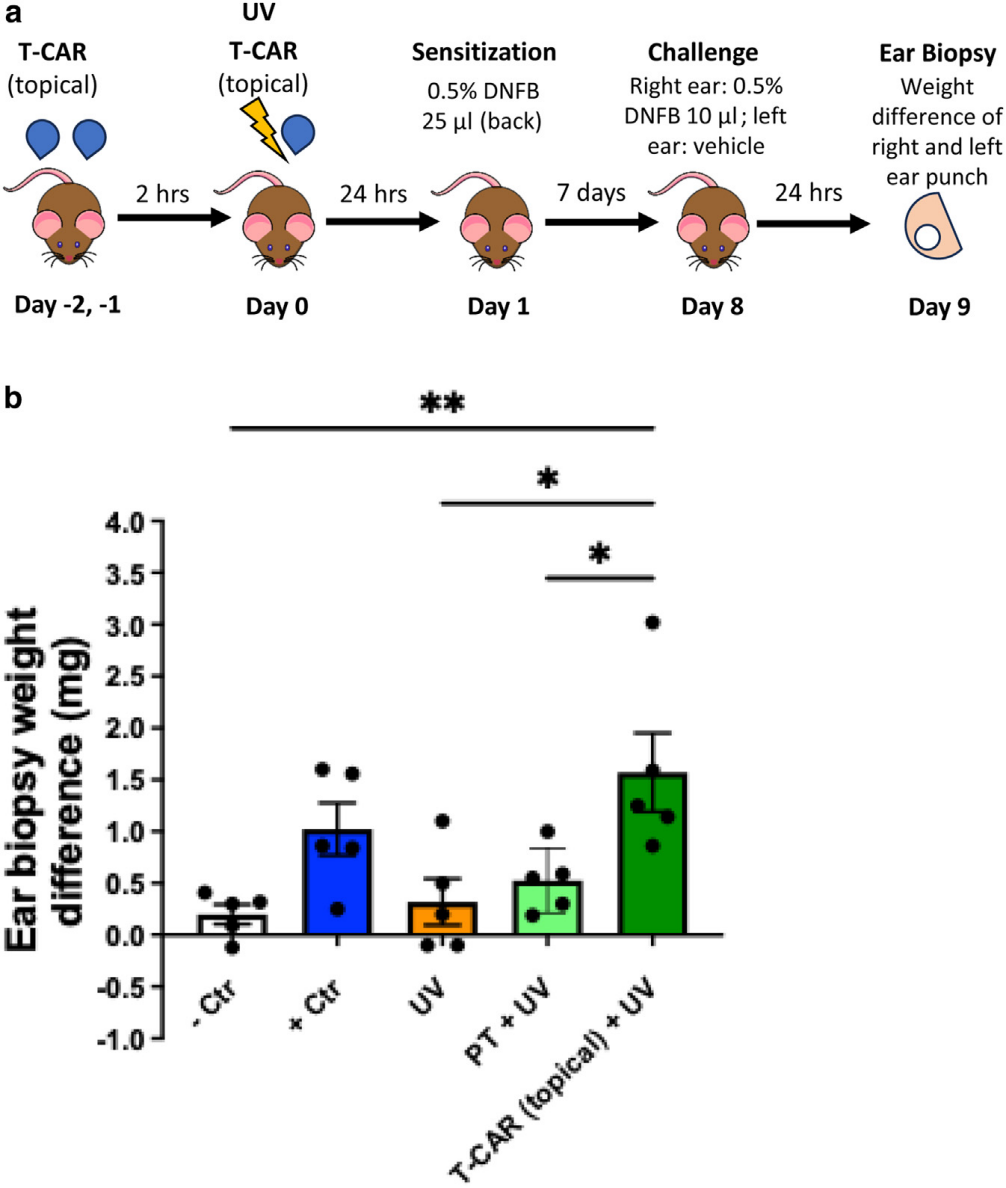
**Topical T-CAR gel prevented UV-induced suppression of CHS reactions.** (**a**) Experimental scheme of CHS assay in the SKH-1 mice. Mice were topically treated with T-CAR gel containing 10 mM carvedilol (200 μl) or PT (the same transfersome gel without drug loading, 200 μl) once a day for 3 days with the third dose right after UV exposure. Twenty-four h after, mice were sensitized with DNFB (25 μl) (0.5% in acetone:olive oil, 4:1, vol/vol) on UV-treated back skin. After 7 days, the right ears were challenged with DNFB (10 μl, 0.5%), and the left ears were treated with vehicle. Twenty-four h after, 5-mm punch biopsies were obtained from both ears and weighed. (**b**) Effects of topically administrated T-CAR (10 mM), in comparison with PT (without drug) on UVR (224 mJ/cm^2^)-induced suppression of CHS reactions in mice (male only). The data shown are the mean ± SEM difference in ear biopsy weights (right ear minus left ear) (n = 5). One-way ANOVA with Tukey posthoc test was used to assess statistical differences. Multiplicity adjusted *P*-values for each comparison are shown: ∗*P* < .05 and ∗∗*P* < .01. CHS, contact hypersensitivity; Ctr, control; DNFB, 2,4-dinitro-1-fluorobenzene; h, hour; PT, plain transfersome; T-CAR, transfersomal carvedilol.

We next examined whether topical administration of carvedilol has an effect similar to that of systemic drug administration. We previously developed and characterized a topical delivery system on the basis of the nanotransfersome technology for carvedilol, namely transfersomal carvedilol **(**T-CAR) (Abdullah Shamim et al, 2022; Chen et al, 2020b). T-CAR encapsulated with 10 mM carvedilol was incorporated into a gel formulation to facilitate topical drug application (Abdullah Shamim et al, 2022). CHS assay was used to evaluate the effects of topically applied T-CAR on UV-induced immunosuppression. The mice were pretreated with daily topical T-CAR 2 days in a row and then subjected to UV exposure on day 0, followed by a third dose of T-CAR after UV exposure (drug treatments on days −1, −2, and 0). To avoid the potential sunscreen effects of carvedilol (Huang et al, 2017), the T-CAR gel was applied immediately after UVR (Figure 3a). Compared with the UV only group, T-CAR treatment was significantly higher in CHS reaction (*P* = .0113), indicating that T-CAR reversed UV-induced CHS suppression. The difference between the plain transfersome group (the empty transfersome gel control without drug) and UV only groups was nonsignificant (*P* = .97) (Figure 3b). In this experiment, the positive control group was not significantly higher than the UV only group (*P* = .27) owing to a high variation in these 2 groups. The topical drug treatment experiment also used male mice, confirming the treatment effects of carvedilol in male mice. Because T-CAR treatment exhibits activity in preventing chronic UVR-induced skin tumor formation in SKH-1 mice (Abdullah Shamim et al, 2022), these data shown in Figure 3 confirm that carvedilol’s skin cancer preventive activity is attributed to its protective effects against UV-induced immunosuppression.

### Carvedilol and its R- and S-enantiomers similarly prevented UV-induced immunosuppression

**Figure 4:**
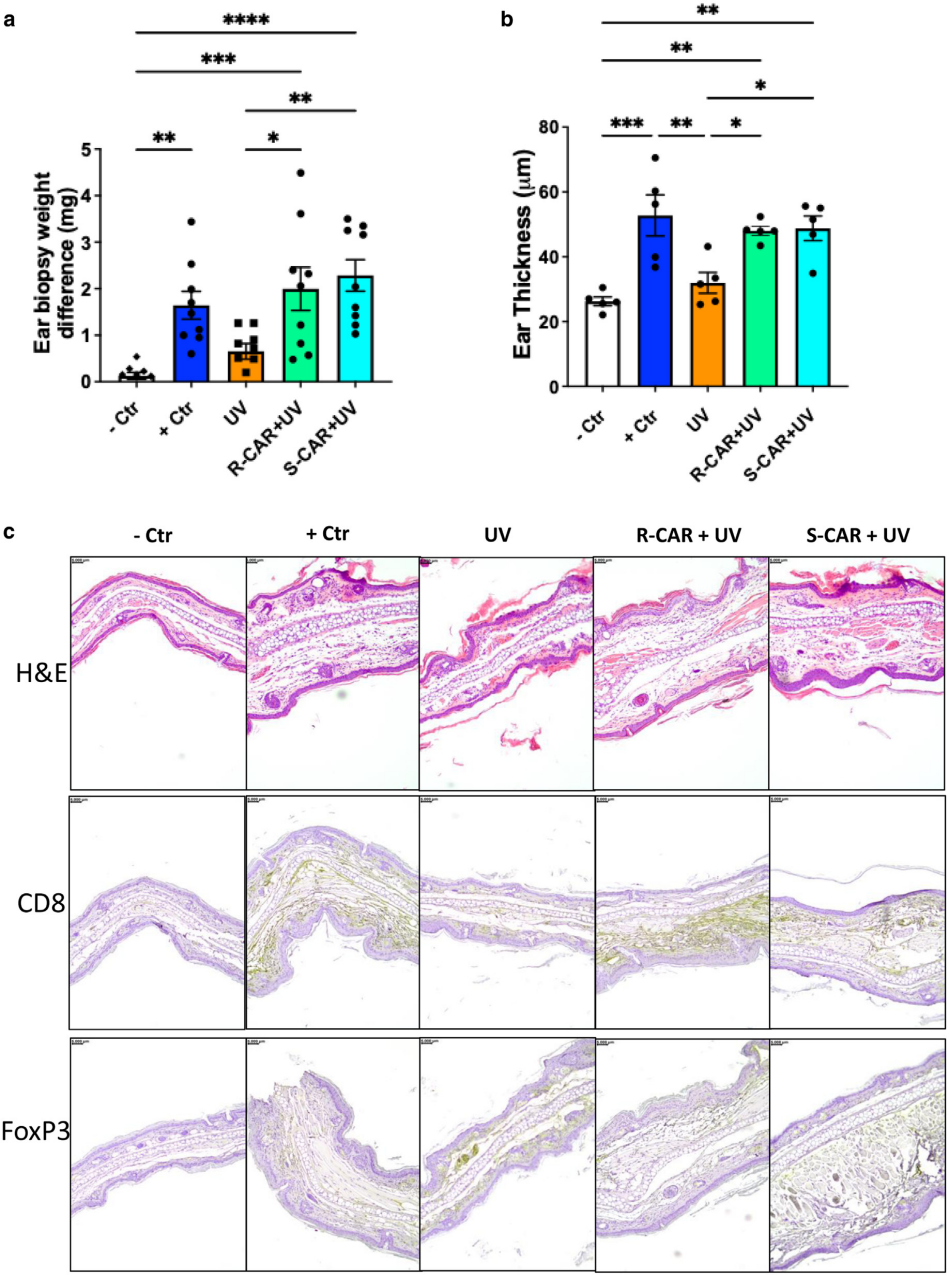
**Systemically administered R- and S-CAR similarly prevented UV-induced suppression of CHS reactions.** (**a**) The mice (male only) were pretreated with R-CAR (10 mg/kg) or S-CAR (10 mg/kg) through i.p. injection 2 h before UVR (224 mJ/cm^2^) on day 0. On day 1, mice were sensitized with DNFB (25 μl) (0.5% in acetone:olive oil, 4:1, vol/vol) on UV-treated back skin. After 7 days, the right ears were challenged with DNFB (10 μl, 0.5%), and the left ears were treated with vehicle. Twenty-four h after, 5-mm punch biopsies were obtained from both ears and weighed. The data shown are the mean ± SEM difference in ear biopsy weights (right ear minus left ear) (n = 10). (**b**) To confirm the ear punch biopsy weight data, the ear thickness was measured under microscope on the H&E-stained ear sections. One-way ANOVA with Tukey posthoc test was used to assess statistical differences. Multiplicity adjusted *P*-values for each comparison are shown: ∗*P* < .05, ∗∗*P* < .01, ∗∗∗*P* < .001, and ∗∗∗∗*P* < .0001. (**c**) Representative microphotographs of mouse ear punch biopsy stained with H&E or stained with primary antibodies of CD8 or FoxP3 for IHC analysis (bar = 5 mm). CAR, carvedilol; CHS, contact hypersensitivity; Ctr, control; DNFB, 2,4-dinitro-1-fluorobenzene; h, hour; IHC, immunohistochemical; i.p., intraperitoneal.

Because carvedilol, a racemic mixture containing R- and S-carvedilol enantiomers in 1:1 ratio, attenuates UV-mediated suppression of CHS response to DNFB in SKH-1 mice (Figures 1 and 2), we next examined whether the pure R- and S-carvedilol enantiomers have the same effect in male SKH-1 mice. As expected, a single-dose intraperitoneally injection of R- or S-carvedilol (10 mg/kg) 2 hours before UV exposure significantly attenuated UV-induced CHS suppression (*P* = .024 and .004, respectively) (Figure 4a). To confirm the ear weight data, the right ears were stained with H&E for histological examination. DNFB challenge of the ear induced considerable edema of the ear in sensitized mice (positive control) compared with that in the negative control (Figure 4b and c). The edema was reduced in UV-exposed mice, whereas both S- and R-carvedilol treatments significantly reversed the UV-induced repression of the ear edema (Figure 4b and c).

Previous studies have indicated that maintaining a balance between CD8+ T cells and Tregs is an important factor in determining the duration and intensity of CHS reactions (Honda et al, 2014; Ring et al, 2006; Vocanson et al, 2010). When CD8+ T cells are predominant, the CHS reaction tends to be more severe and prolonged (Ring et al, 2006). On the other hand, a higher proportion of Tregs helps to suppress the immune response (Vocanson et al, 2010). Using immunohistochemical analysis, we found that a larger number of CD8+ cells were attracted to the ears after DNFB challenge in mice treated with R- and S-carvedilol than in those treated with UV alone (Figure 4c). Conversely, the number of FoxP3 staining, which serves as a marker for Tregs, was decreased in the ears of R- and S-carvedilol−treated mice compared with that in those treated with UV alone. These findings suggest that UV suppresses the immune system by increasing Tregs and decreasing CD8+ cells, whereas R- and S-carvedilol can reverse the suppressing effects of the UV.

### UV induced immunosuppression in β2-AR–KO mice

**Figure 5:**
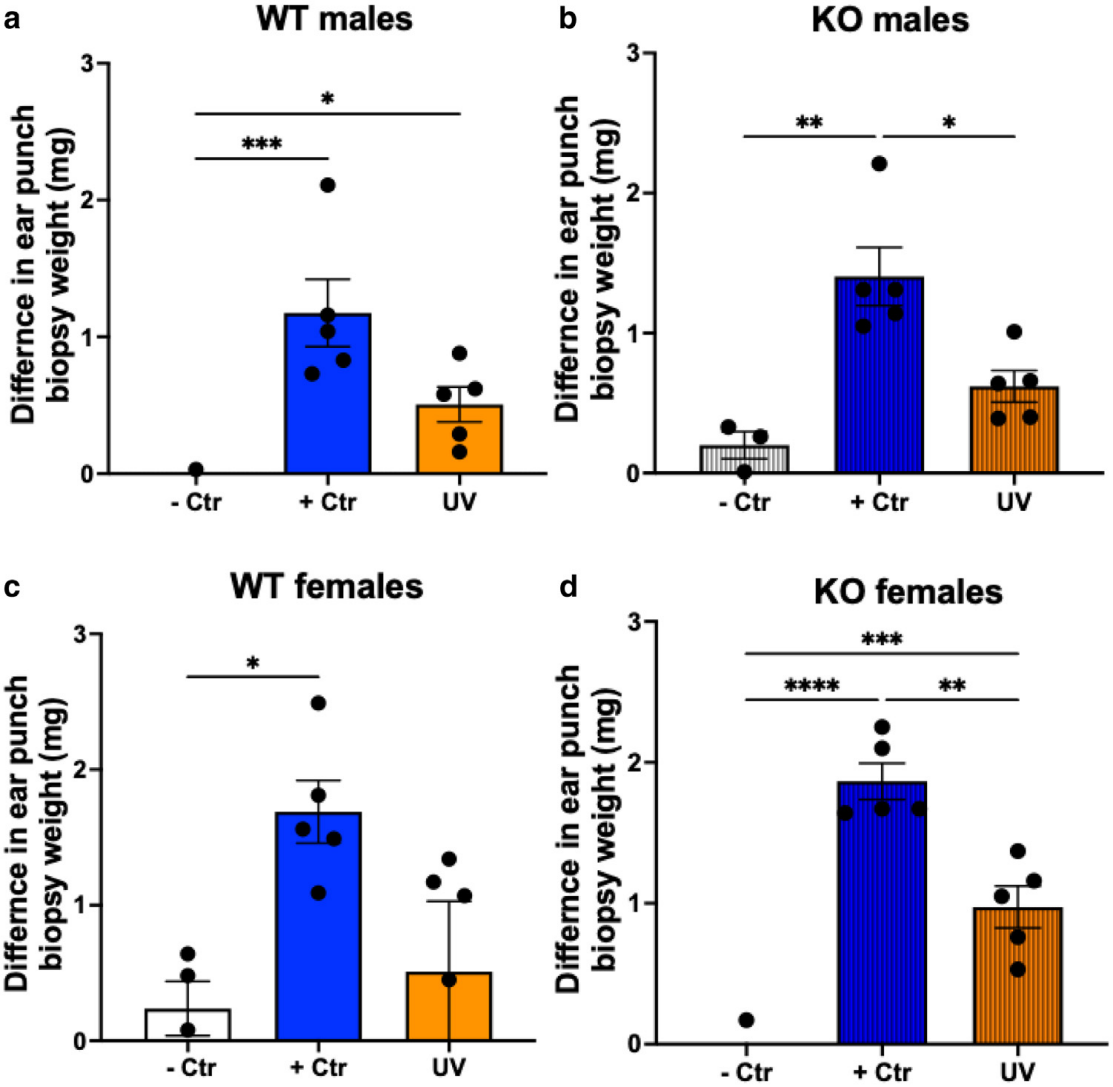
**Effect of β2-AR (*Adrb2*) gene deletion on UV-induced suppression of CHS in SKH-1 mice.** The KO and WT (male and female) mice were treated with single-dose UVR (224 mJ/cm^2^) on day 0 before being sensitized to DNFB on day 1, and elicitation reactions (challenge) were performed on the dorsal ears on day 8. On day 9, 5-mm punch biopsies were obtained from both ears and weighed. (**a, b**) Showing the difference in ear punch biopsy weight (mg) 24 h after the challenge in male mice of WT and KO, respectively. (**b, c**) The difference in ear punch biopsy weight (mg) 24 h after the challenge in female mice of WT and KO, respectively. The data listed are the mean ± SEM of difference in ear punch biopsy weight (right left) from n = 5 mice. One-way ANOVA with Tukey posthoc test was used to assess statistical differences. Multiplicity adjusted *P-*values for each comparison are shown: ∗*P* < .05, ∗∗*P* < .01, ∗∗∗*P* < .001, and ∗∗∗∗*P* < .0001. β2-AR, β2-adrenergic receptor; CHS, contact hypersensitivity; Ctr, control; DNFB, 2,4-dinitro-1-fluorobenzene; h, hour; KO, knockout; WT, wild-type.

To examine the role of β2-AR in UV-induced immunosuppression, we established a mouse model of whole-body β2-AR gene (*Adrb2*) deletion on the SKH-1 background. The WT SKH-1 mice and the KO SKH-1 mice were subjected to UV exposure and the CHS protocol (Figure 1a), in comparison with the positive and negative controls. As can be seen from Figure 5, UVR suppressed CHS reactions in WT and KO mice. The experiments were conducted in both males and females, and similar results were obtained with different degrees of significance: UV’s effect in WT males *P* = .052, UV’s effect in KO males *P* = .012, UV’s effect in WT females *P* = .091, and UV’s effect in KO females *P* = .001. Thus, the immunosuppressing effects of UV in KO animals were more pronounced. Although previous studies reported that injection of catecholamines suppressed CHS reactions in mice (Seiffert et al, 2002) and that β2-AR may play a role in cutaneous immunity, the data obtained in β2-AR–KO mice indicate that β2-AR is dispensable for UV-induced immunosuppression. Future studies should evaluate whether endogenous and exogenous catecholamines can affect CHS reactions in the WT and KO mice.

### Carvedilol treatment prevented UV-induced immunosuppression in β2-AR–KO mice

**Figure 6:**
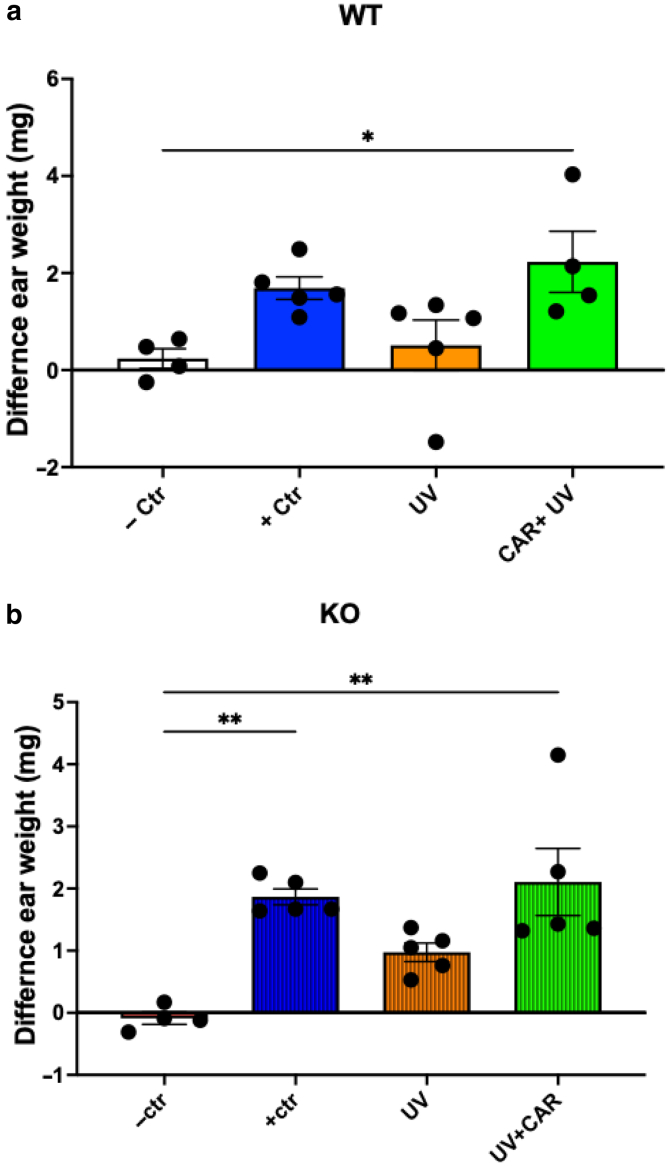
**Effects of carvedilol treatment on UV-induced immunosuppression in WT and β2-AR–KO mice.** The (**a**) WT or (**b**) KO mice (female only) were pretreated with CAR (10 mg/kg, i.p.) 2 h before UV (224 mJ/cm^2^) radiation on day 0 before being sensitized to DNFB on day 1, and elicitation reactions (challenge) were performed on the dorsal ears on day 8. On day 9, 5-mm punch biopsies were obtained from both ears and weighed. The data are mean ± SEM difference in ear biopsy weights (right left) (n = 4 for negative controls; n = 5 for other groups). One-way ANOVA with Tukey posthoc test was used to assess statistical differences. Multiplicity adjusted *P-*values for each comparison are shown: ∗*P* < .05 and ∗∗*P* < .01. β2-AR, β2-adrenergic receptor; CAR, carvedilol; Ctr, control; DNFB, 2,4-dinitro-1-fluorobenzene; h, hour; i.p., intraperitoneal; KO, knockout; WT, wild-type.

We next determined whether β2-AR is involved in carvedilol’s activity against UV-induced immunosuppression using the β2-AR–KO mice (female mice only for this experiment). Systemic administration of carvedilol (10 mg/kg, intraperitoneally) 2 hours before the UV exposure was performed on the WT and KO mice. In this experiment, owing to a small sample size, UV exposure did not significantly reduce CHS reactions compared with that in the positive control groups (*P* = .22 and .20 for WT and KO, respectively). Even though the suppressing effects of UV were modest in mice of both genotypes, carvedilol treatment reversed UV-induced CHS suppression in both WT and KO mice (*P* = .061 and .074 for WT and KO, respectively) (Figure 6). These data indicate that carvedilol prevents UV-mediated immunosuppression independently of the presence of β2-AR.

### Carvedilol prevented UV-induced skin carcinogenesis in both WT and β2-AR–KO mice

**Figure 7:**
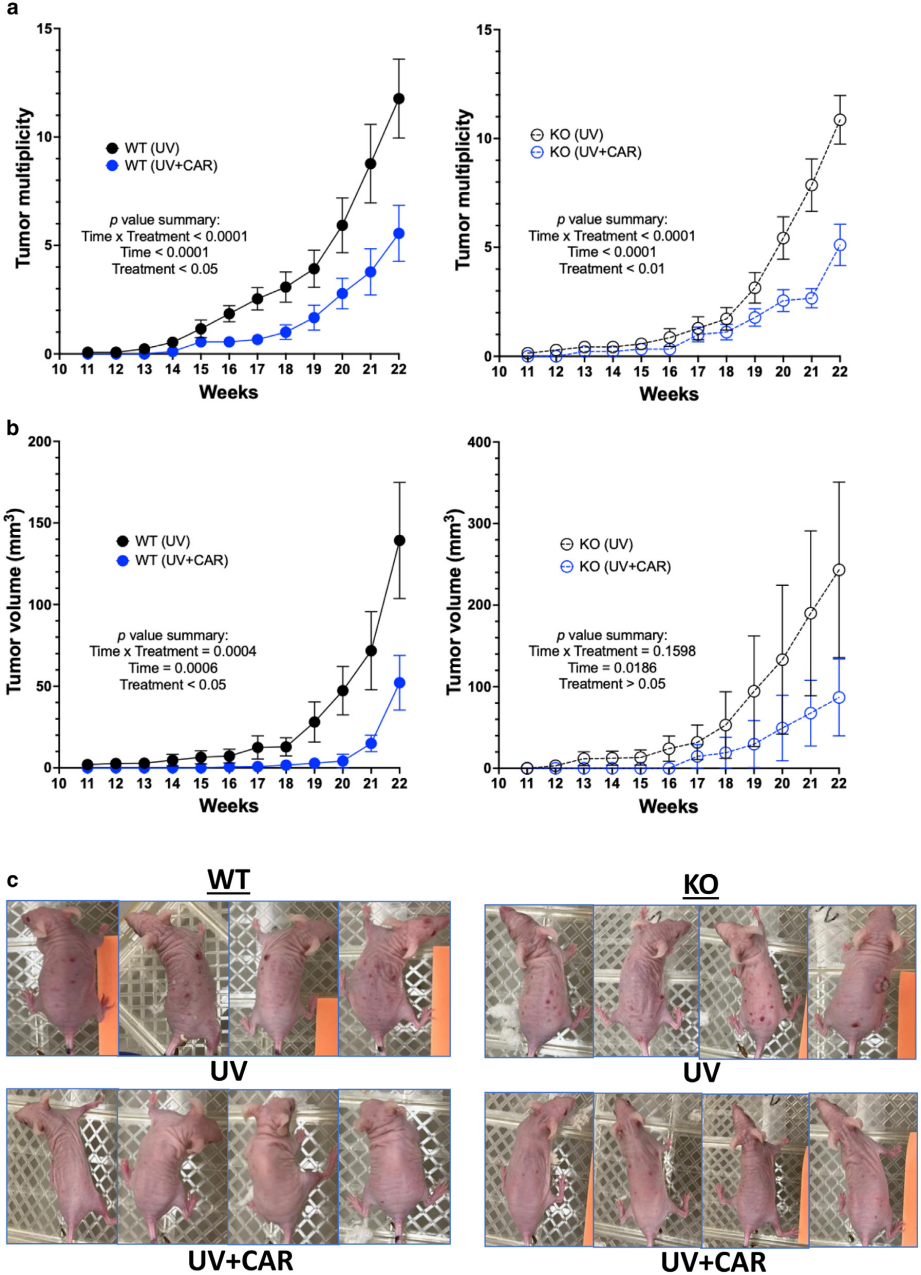
**Effects of CAR on the development of UV-induced skin tumors in WT and β2-AR–KO mice.** Mice (female only) were pretreated with 10 μM CAR dissolved in acetone 3 times per week for 2 weeks. Then, the mice were exposed to UV 3 times per week, and drug treatments were given right after each irradiation. (**a**) Tumor multiplicity (average number of tumors per mouse) in the WT (left) and KO (right) mice with (blue) or without CAR treatment (black). (**b**) Average tumor volume per mouse in the WT and KO mice with or without CAR treatment. The data are mean ± SEM (n = 13 in the WT control group; n = 9 in the CAR/WT group; n = 7 in the KO control group; n = 9 in the CAR/KO group). The data were analyzed with repeated-measures 2-way ANOVA with the Geisser–Greenhouse correction. The *P*-values are shown in individual plots. (**c**) Representative photographs of mice from the UV-treated and UV + CAR groups. β2-AR, β2-adrenergic receptor; CAR, carvedilol; KO, knockout; WT, wild-type.

To address the question of whether carvedilol prevents skin cancer by targeting the β2-AR, we next examined the effects of topical carvedilol (10 mM) treatment on UV-induced skin carcinogenesis in the WT and KO SKH-1 mice. Both the WT and KO mice developed tumor with the same multiplicity (*P* = .53) and had no statistical difference in tumor volume (*P* = .20). As can been seen from Figure 7, carvedilol significantly reduced the tumor multiplicity in both WT and KO mice (Figure 7a). Carvedilol significantly reduced tumor volume in the WT mice but not in the KO mice. The reduction of tumor volume in the KO mice was not significant owing to a high variation of tumor sizes in the KO mice (Figure 7b). These data confirm our hypothesis that carvedilol’s anticancer effects were independent on its b-blocking activity.

**Figure 8:**
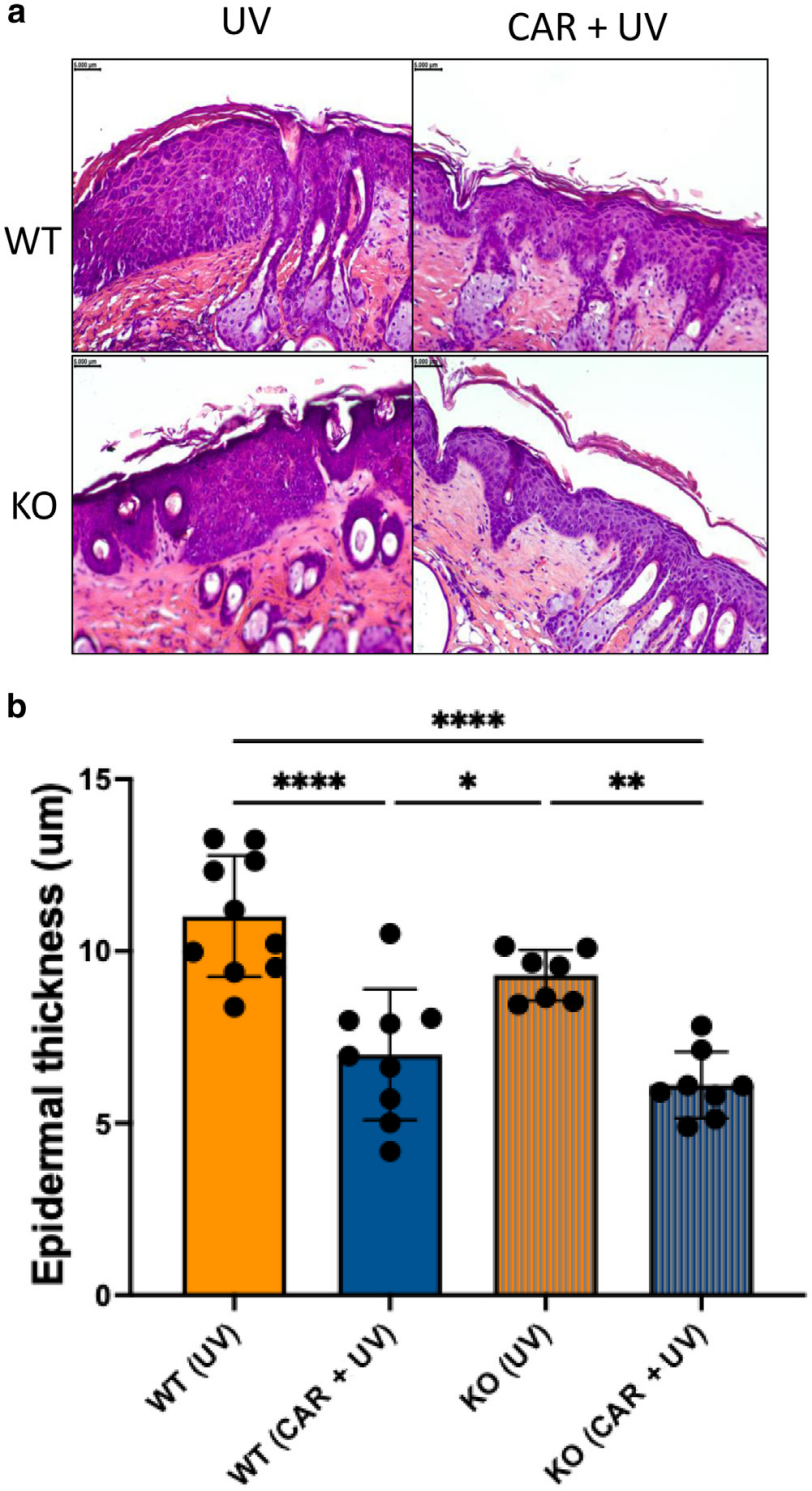
**Effects of CAR on chronic UV-induced skin thickening in WT and β2-AR–KO mice.** Mice were pretreated with 10 μM CAR dissolved in acetone 3 times per week for 2 weeks. The mice were then exposed to UV 3 times per week, and drug treatments were given right after each irradiation. (**a**) Representative H&E staining of nontumor skin sections obtained from WT and KO mice after UV and/or CAR treatment (bar = 5 mm). (**b**) To quantify epidermal thickness in nontumor skin section, 2 images were collected from each mouse, and in each image, the thickness was measured 3 times and averaged to get the final data points for each mouse. The data are mean ± SEM from 10 mice per group. One-way ANOVA with Tukey posthoc test was used to assess statistical differences. Multiplicity adjusted *P*-values for each comparison are shown: ∗*P* < .05, ∗∗*P* < .01, and ∗∗∗∗*P* < .0001. β2-AR, β2-adrenergic receptor; CAR, carvedilol; KO, knockout; WT, wild-type.

**Figure 9:**
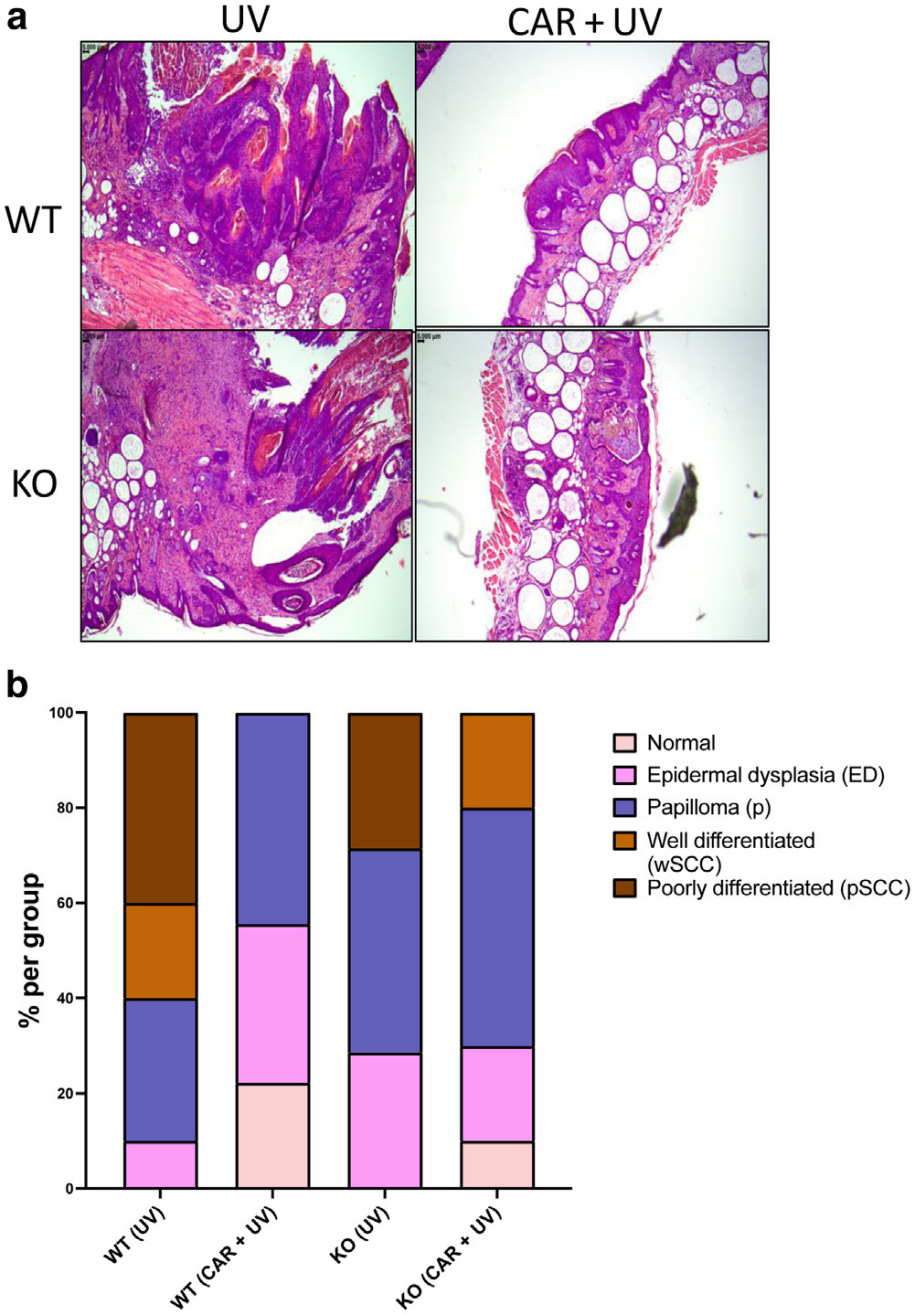
**Effects of CAR on chronic UV-induced skin tumors in WT and β2-AR–KO mice.** Mice were pretreated with 10 μM CAR dissolved in acetone 3 times per week for 2 weeks. The mice were then exposed to UV 3 times per week, and drug treatments were given right after each irradiation. (**a**) Representative H&E staining of tumor sections obtained from WT and KO mice after UV and/or CAR treatment (bar = 5 mm). (**b**) Skin tumor histological analysis. The skin lesions were classified as normal, ED, papilloma (denoted as p), wSCC, and pSCC. β2-AR, β2-adrenergic receptor; CAR, carvedilol; *ED,* epidermal dysplasia; KO, knockout; pSCC, poorly differentiated squamous cell carcinoma; wSCC, well-differentiated squamous cell carcinoma; WT, wild-type.

Histological analysis of nontumorous skin sections isolated from mice treated with only UV or CAR + UV in both WT and KO is displayed in Figure 8. UV induced a strong increase of epidermal thickness in both the WT and KO groups; however, treatment with carvedilol resulted in a significant decrease in epidermal thickness (*P* < .0001 for the WT group and *P* = .001 for the KO group). There was no significant difference between the WT (UV) and KO (UV) mice (*P* = .11). Upon UV exposure, the severity of skin tumors was higher in UV-treated control mice than in those treated with carvedilol in both WT and KO mice (Figure 9). At the end of treatment (week 22), the WT UV control group had mice with 40% poorly differentiated squamous cell carcinoma (SCC) and 20% well-differentiated SCC, but carvedilol-treated WT mice had no SCC but only benign papilloma or epidermal dysplasia. The KO UV control group had 29% poorly differentiated SCC, whereas KO carvedilol group had no poorly differentiated SCC and 20% well-differentiated SCC. These data indicate that carvedilol showed treatment effects in both WT and KO mice.

## Discussion

In this study, the β-blocker carvedilol demonstrated activity in preventing UV-induced immunosuppression independently of β-blockade. This conclusion is supported by the following data: (i) both β-blocking S-carvedilol and non–β-blocking R-carvedilol enantiomers attenuated UV-induced immunosuppression in SKH-1 hairless mice, (ii) UV suppressed CHS reactions in both β2-AR–KO and WT mice, and (iii) carvedilol treatment attenuated UV-induced immunosuppression in both genotypes. Furthermore, the carcinogenesis data confirm that carvedilol prevents UV-induced skin tumor formation independently of β-blockade because both WT and KO mice exposed to chronic UVRs developed skin tumors, and carvedilol treatment inhibited skin tumor development in both genotypes. Taken together, these data indicate that carvedilol’s skin cancer preventive activity is attributed to its activity against UV-induced immunosuppression through a mechanism independent of its known activity of β-adrenergic blockade. Because the effects of UV on the immune system play an important role in leading to cancer development in the skin (Bernard et al, 2019), the preventive effects of carvedilol against UV-induced skin carcinogenesis may be attributed to its effects on immunosuppression.

Effects of carvedilol on UV-induced immunosuppression were examined using a classical CHS assay in response to the contact allergen DNFB in SKH-1 hairless mice. Although SKH-1 is a commonly used strain in skin carcinogenesis studies (Benavides et al, 2009), this strain is not commonly used for CHS assay. Therefore, we first evaluated 2 UV doses and found that in the SKH-1 strain, a single dose of UVR (224 mJ/cm^2^) significantly suppressed the CHS response to DNFB (Figure 1), and therefore, this dose was used throughout this study. Systemic administration of a single dose of carvedilol (intraperitoneal injection) at the dose of 10 mg/kg, applied 2 hours before the UV exposure, significantly reversed UV-mediated CHS suppression (Figure 1). Carvedilol’s effects were confirmed by an alternative route of drug administration, that is, topical application on the mouse skin with a nanotransfersome delivery system that was characterized previously (Abdullah Shamim et al, 2022) (Figure 3).

Carvedilol’s effects on immunosuppression were confirmed by the adoptive transfer of lymphocytes isolated from the skin-draining lymph nodes and spleens of UV-irradiated mice with or without carvedilol treatment (Figure 2). Previous studies have shown that UV affects primarily T-cell–driven immune reactions, and UV-induced immunosuppression is antigen specific, owing to the induction of antigen-specific T cells with suppressive features, that is, Tregs (Schwarz, 2010). Injection of lymphocytes obtained from UV- and DNFB-treated mice into naïve mice renders the recipients nonresponsive to DNFB sensitization. Using the adoptive transfer experiments, we demonstrated that carvedilol was able to prevent the immunosuppressing effects of UV on the donor and recipient mice (Figure 2). Flow cytometry analysis confirms that carvedilol treatment reduced the number of Tregs. UV mediated immunosuppression through multiple mechanisms, involving many components of the immune system (innate and adaptive immune systems) and soluble factors. Carvedilol may target Tregs directly or indirectly by affecting Treg production or function. Future studies should further determine the effects of carvedilol on various components of the immune system.

Results from this study are consistent with previous findings that R- and S-carvedilol similarly inhibited UV-induced skin carcinogenesis in SKH-1 mice (Liang et al, 2021). This investigation used β2-AR–KO mice on the SKH-1 background to decipher the role of this receptor in UV-mediated reactions. The KO mice and the WT littermate controls of the SKH-1 hairless strain offer a good opportunity to study UV effects because hair blocks UV, and pigmentation also affects UV response (Benavides et al, 2009). β-Adrenergic receptors, particularly β2-AR, have been shown to play a role in cancer development and anticancer immunity (Qiao et al, 2021). Several studies reported the use of β2-AR–KO mice. For example, the β2-AR–KO mice have been used to examine the role of β2-AR and adrenergic stress signaling in a syngeneic mouse model of breast cancer (4T1) (Bucsek et al, 2017). In the breast cancer study, the β2-AR–KO mice at the BALB/c background and the β-blocker propranolol demonstrated an increased intratumor frequency of CD8^+^ T cells with an effector phenotype and an increased efficacy of anti–PD-1 checkpoint blockade. This study concludes that the antitumor effects of propranolol depend on β-blockade because its tumor inhibitory activity was lost in the β2-AR–KO mice (Bucsek et al, 2017). In fact, our initial hypothesis was that carvedilol prevents skin cancer by targeting β2-AR (the main isoform expressed in the epidermal keratinocytes and immune cells). However, the data we have obtained so far collectively suggest an opposite hypothesis: the skin cancer preventive activity of carvedilol is not attributed to its β-blocking property. The discrepancy regarding the involvement of β2-AR in different types of cancer could be due to different models used. This study used a de novo tumor model, which is induced using a complete carcinogen (UV) in healthy mice. The data obtained from this study show that the WT and β2-AR–KO mice demonstrated similar responses to UV on the basis of the CHS assay (Figure 5) and skin carcinogenesis induced by chronic UV (Figure 7). However, we could not completely rule out the contribution of β2-AR because our experiments were conducted in animals without stress or other factors that activate the β2-AR. Stress may be an important contributor because Gillis et al (2021) demonstrated that carvedilol had no effect on the growth of MDA-MB-231 xenografts in nonstressed mice but had a markedly inhibitory effect when the animals were exposed to restraint stress. In the future, repeating the study with stressed animals of β2-AR–KO and WT genotypes is needed to confirm the role of β2-AR in UV-induced immunosuppression and carcinogenesis.

One possible target for carvedilol’s immunomodulatory activity in mice is the AhR. AhR has been previously associated with UV-induced immunosuppression because the AhR agonists suppressed the induction of CHS reactions and induced antigen-specific Tregs similar to the effects of UV, and UV-induced immunosuppression was reduced in the *AhR* gene–KO mice (Navid et al, 2013). As a ligand-activated transcription factor, AhR can be activated by multiple ligands, including benzo(a)pyrene and UV-induced FICZ (Congues et al, 2024). A previous study showed that carvedilol was able to inhibit benzo(a)pyrene-induced AhR activity and reduce AhR-regulated mRNA expression of *CYP1A1* (Shahid et al, 2023). Therefore, carvedilol may inhibit UV-induced immunosuppression by inhibiting UV-activated AhR signaling. Further studies are needed to investigate whether AhR is the target for carvedilol’s skin cancer preventive activity.

The protocol we applied for the CHS assay was based on a single-dose UVR, whereas most published work is based on a protocol of administering UV daily for 4 consecutive days (Bruhs and Schwarz, 2017). Furthermore, only local immunosuppression is evaluated in this study, where the hapten DNFB was applied topically to the same area irradiated by the UV (Miyauchi and Horio, 1995). Future studies should evaluate the effects of carvedilol on UV-induced systemic immunosuppression. In addition, only a single dose of carvedilol was examined: intraperitoneal injection at the dose of 10 mg/kg or topical administration at the dose of 10 mM. Future studies should examine dose-dependent effects of carvedilol and its enantiomers.

In summary, this study indicates that β2-AR is dispensable in UV-induced immunosuppression as well as in the protective activity of carvedilol. These data also indicate that carvedilol prevents UV-induced skin cancer by reversing UV-mediated immunosuppression.

## Materials and Methods

### Chemicals

Carvedilol was purchased from Santa Cruz Biotechnology. The optically pure R-and S-carvedilol were synthesized at Chem-Impex International and verified by chiral HPLC using Phenomenex Lux 5 μm Cellulose-4 LC Column 250 × 4.6 mm (Phenomenex). These compounds were reconstituted in DMSO (5.0 mg/ml). The allergen DNFB was purchased from Sigma-Aldrich.

### Animal models

All animal studies were carried out under the recommendations and guidelines established by the Western University of Health Sciences’ Institutional Animal Care and Use Committees. Mice had access to water and food ad libitum and were housed on a 12-hour light/dark cycle with 35% humidity. To establish the β2-AR–KO mouse model on the SKH-1 genetic background, mice that were homozygous null for both β1-andrenergic receptor and β2-AR, encoded by *Adrb1* and *Adrb2* genes, respectively, were purchased from Jackson Laboratory (Adrb1^tm1Bkk^ Adrb2^tm1Bkk^/J, stock number 003810) (Rohrer et al, 1999). The double KO mice were bred with C57BL/6J mice, which were purchased from Jackson Laboratory, to generate a heterozygous F1 generation. The F1 generation was bred together to generate the F2 generation, of which one sixteenth of the offspring was a β2-AR single gene–KO mouse. To obtain the hairless β2-AR–KO mice, SKH-1 mice, which were purchased from Charles River, were bred with the β2-AR–KO mice for more than 10 rounds to generate hairless β2-AR–KO mouse and WT littermates that were on SKH-1 background.

### UV light source and animal exposure procedure

The UV lamps emitting UVB (280–320 nm; 54% of total energy), UVA (320–400 nm; 37% of total energy), UVC (100–280 nm; 2% of total energy), and visible light (400–450 nm; 7% of total energy; catalog numbers 95-0042-08 and 95-0043-13, UVP, Upland, CA) were used in all experiments. Stable power output (mW/cm^2^) was measured using an UVX Radiometer (number 97-0015-02, UVP) coupled with a sensor with a calibration point of 310 nm (UVX-31, number 97-0016-04, UVP), and exposure time was calculated using the following formula: dose (mJ/cm^2^) = exposure time (second) × output intensity (mW/cm^2^). Only the UVB output was measured because UVB range is the primary source of immunosuppression (Schwarz, 2005), so the doses refer to the UVB component only. Quality control of the lamps and exposure time were calculated and monitored each time before using the lamps to account for power output changes. During UV exposure, mice roamed freely in acrylic cages on a rotating platform, ensuring consistent and equal dorsal distribution of UV irradiation. The distance from the lamps to the mice was approximately 30 inches. The UV light source used in this study does not mimic the UV components of natural sunlight (including 95% UVA and 5% UVB along with visible and infrared light). Future studies using natural sunlight in real-world conditions can improve clinical relevance.

### UV-induced immunosuppression of CHS

The immunosuppressive effects of UV have been demonstrated by a classical CHS reaction to allergens such as the hapten DNFB. CHS is primarily a T-cell–mediated immune response inhibitable by UV (mainly the UVB component) exposure (Bruhs and Schwarz, 2017). We applied a modified CHS protocol by the use of single-dose UV and measuring the weight of ear punch biopsy (Sahu et al, 2012) (Figure 1a). Ear punch biopsies were weighted because the data obtained on the basis of ear punch biopsy weight were more reproducible than measurement of ear thickness with callipers (Sahu et al, 2012). Drugs (carvedilol, R-carvedilol, or S-carvedilol) were administered by intraperitoneal injection, 2 hours before UV exposure. On day 0, the backs of the mice were exposed to a single-dose UVR at the dose of 120 mJ/cm^2^ or 224 mJ/cm^2^. Twenty-four hours after UVB exposure (day 1), the mice were sensitized with 25 μl freshly prepared DNFB (0.5% in acetone:olive oil, 4:1, v/v) administered to the back of the mice. Seven days later (day 8), the mice were challenged with 10 μl of 0.5% DNFB solution on the right ear skin (dorsal side only), whereas the left ear was treated with vehicle (acetone:olive oil, 4:1, v/v). After 24 hours (day 9), the mice were killed; 5-mm punch biopsies were obtained from the ears and weighed and stored in 10% formalin for histological analyses. Non–UV-irradiated mice that received the same doses of DNFB served as a positive control. Non–UV-irradiated mice that received only ear challenges served as a negative control. For topical drug application, transfersome containing 10 mM carvedilol (T-CAR) or the plain transfersome as vehicle control (volume 200 μl) was applied topically to the back of the mice (the same area of UV exposure and DNFB sensitization) daily for a total of 3 treatments. Development and characterization of the T-CAR formulation was described previously (Abdullah Shamim et al, 2022; Chen et al, 2020b). To avoid sunscreen effects, the third dose was given immediately after UVR.

### Adoptive cell transfer assay

Previous studies indicate that UV induces immunosuppression primarily by inducing the overproduction and increased activity of antigen-specific Tregs (Schwarz, 2005). Therefore, UV-induced immunosuppression can be adoptively transferred by injecting lymphocytes isolated from the spleen and skin-draining lymph nodes, which contain Tregs, from UV-irradiated mice into naive recipients without UV exposure. The procedure has been described in previous reports (Navid et al, 2013) (Figure 2a). In brief, the donor mice were divided into 2 groups (n = 10) treated with vehicle or carvedilol (10 mg/kg) (intraperitoneally) 2 hours before a single dose of UVR (224 mJ/cm^2^). Twenty-four hours after UV exposure, the mice were sensitized with DNFB, as described previously. Five days later, mice were killed for collection of spleen and the inguinal, axillary, and brachial lymph nodes, which were dissected, separated into single-cell suspensions, and placed into RPMI complete medium and pooled into 2 groups. The lymph node cells and splenocytes were obtained from the UV and CAR + UV groups, respectively, and injected intravenously (5 × 10^7^ ∼ 1 × 10^8^ lymphocytes in 200 μl of saline per mouse) into naive mice (adoptively transferred) 24 hours before DNFB sensitization. After 7 days, ears were challenged with DNFB in the right ear, and ear swelling was measured as described previously. Non–UVB-irradiated mice that received the same dose of DNFB served as a positive control. Non–UVB-irradiated mice that received only ear challenge served as a negative control. UV-irradiated mice were included for comparison. For flow cytometry analysis, the single-cell suspensions of lymphocytes were obtained from UV and CAR + UV groups (n = 3) using the same method described earlier. The cell suspension was centrifuged at 300*g* for 5 minutes. After this, the pellet was resuspended and diluted at a ratio of 1:10 using 1× Red Blood Cell Lysis Solution (Miltenyi Biotec). The mixture was vortexed for 5 seconds and then incubated at room temperature for 2 minutes. After incubation, the cells were centrifuged again at 300*g* for 5 minutes. The resulting cell pellet was resuspended in a flow cytometry buffer, and the cells were counted to achieve a concentration of 1 × 10^6^ cells. To block Fc receptors, the cells were preincubated with TruStain FcX PLUS for 10 minutes on ice. The cells were subsequently stained with SB780 antimouse CD4 and SB 645 antimouse CD25 antibodies (Thermo Fisher Scientific) and incubated at 4 °C for 30 minutes. After staining, the cells were washed twice with buffer. The supernatant was discarded after the final wash. Finally, the samples were analyzed using the Attune Flow Cytometer (Thermo Fisher Scientific).

### Chronic UV-induced murine skin tumorigenesis studies

A chronic UV experiment was conducted to evaluate the effects of carvedilol on UV-induced skin carcinogenesis in WT and KO mice. A total of 40 female SKH-1 mice aged 7–12 weeks were randomly divided into 2 groups per genotype on the basis of body weight (n = 7∼13). Only female mice were used because male mice are more likely to fight with each other, leading to unwanted skin injury. Mice were treated topically with acetone as vehicle or carvedilol dissolved in acetone (10 mM) 3 times per week for 2 weeks before the first dose of UVR. After pretreatment, the mice were irradiated with gradually increasing levels of UV 3 times a week for 22 weeks with an initial dose of 50 mJ/cm^2^ that was increased each week by 25 to 150 mJ/cm^2^, which was continued for the duration of the experiment. All the mice were exposed to UV at the same time. Beginning at the week 11, mice were monitored for tumor development. The time of appearance of the first tumor (latency period) for each mouse was recorded. A tumor is defined as a mass having a diameter of at least 1 mm. Tumors were counted and measured weekly. Two longest measurements (mm) in perpendicular directions were made using a digital calliper and multiplied to obtain a representation of tumor area (expressed as mm^2^). The tumor volume was calculated according to the formula: (width) ^2^ × length/2. The topical drug treatments were continued after UV started on the same day of UVR, immediately after each UV exposure.

### Histology and immunohistochemistry analysis

Formalin-fixed skin tissues were embedded in paraffin, sectioned at 5-μm thickness, and mounted on slides. The slides were then deparaffinized with xylene and rehydrated with ethanol. H&E staining was used to visualize tissue architecture using a Leica DM750 LED Biological Microscope. To calculate epidermal thickness in nontumor skin section, 2 images were collected from each mouse, and in each image, the thickness was measured 3 times and averaged to get the final data points for each mouse. In immunohistochemical analysis, sections were boiled in an antigen retrieval buffer (Abcam, catalog number ab93678) for 20 minutes. Different expression of proteins in tissue was determined using the Vectastain kit, according to the manufacturer’s instructions. In brief, sections were incubated with bloxall endogenous enzyme-blocking solution for 10 minutes to quench endogenous peroxidase activity and then rinsed 3 times (5 minutes each) with Tris-buffered saline with Tween-20 (0.05% Tween-20). Blocking solution was applied for 20 minutes, and then, sections were incubated with diluted antibodies, anti-CD8 (Cell Signaling Technology, 1:400) and anti-FoxP3 (Cell Signaling Technology, 1:400), overnight at 4 °C in a humid chamber. Further processing was performed according to the instructions of the Detection System. Skin sections were evaluated using a Leica DM750 LED Biological Microscope.

### Statistical analysis

All plots were made and analyzed using GraphPad Prism, version 10 (GraphPad Software). Data are presented using individual data points and the mean ± SEM unless stated otherwise. Normality was tested on the basis of Shapiro–Wilk test.

Statistical analysis was performed using 1-way ANOVA followed by a Tukey’s method for multiple comparisons. Repeated-measures 2-way ANOVA with the Geisser–Greenhouse correction was used to analyze longitudinal data, considering time and treatment as fixed effects. For all statistical analyses, a *P* < .05 was considered statistically significant.

## Ethics Statement

All animal studies were approved and carried out under the recommendations and guidelines established by the Western University of Health Sciences’ Institutional Animal Care and Use Committees.

## Data Availability Statement

The data that support the findings of this study are available from the corresponding author (yhuang@westernu.edu) upon request.

## Conflict of Interest

YH, AS, and BTA have United States Utility Patent Application (serial number 17/676,684) filed on February 21, 2022 for the application of carvedilol and R-carvedilol in cancer chemoprevention. The remaining author states no conflict of interest.

## ORCIDs

Ayaz Shahid: http://orcid.org/0000-0002-4120-0557

Fanglong Dong: http://orcid.org/0000-0003-2916-9291

Bradley T. Andresen: http://orcid.org/0000-0002-3948-7456

Ying Huang: http://orcid.org/0000-0003-4756-7939

## References

[bib1] Abdullah Shamim M., Yeung S., Shahid A., Chen M., Wang J., Desai P. (2022). Topical carvedilol delivery prevents UV-induced skin cancer with negligible systemic absorption. Int J Pharm.

[bib2] Benavides F., Oberyszyn T.M., VanBuskirk A.M., Reeve V.E., Kusewitt D.F. (2009). The hairless mouse in skin research. J Dermatol Sci.

[bib3] Bernard J.J., Gallo R.L., Krutmann J. (2019). Photoimmunology: how ultraviolet radiation affects the immune system. Nat Rev Immunol.

[bib4] Bruhs A., Schwarz T. (2017). Ultraviolet radiation-induced immunosuppression: induction of regulatory T cells. Methods Mol Biol.

[bib5] Bucsek M.J., Qiao G., MacDonald C.R., Giridharan T., Evans L., Niedzwecki B. (2017). β-adrenergic signaling in mice housed at standard temperatures suppresses an effector phenotype in CD8^+^ T cells and undermines checkpoint inhibitor therapy. Cancer Res.

[bib6] Calò L.A., Semplicini A., Davis P.A. (2005). Antioxidant and antiinflammatory effect of carvedilol in mononuclear cells of hypertensive patients. Am J Med.

[bib7] Chang A., Yeung S., Thakkar A., Huang K.M., Liu M.M., Kanassatega R.S. (2015). Prevention of skin carcinogenesis by the β-blocker carvedilol. Cancer Prev Res (Phila).

[bib8] Chen M., Liang S., Shahid A., Andresen B.T., Huang Y. (2020). The β-blocker carvedilol prevented ultraviolet-mediated damage of murine epidermal cells and 3D human reconstructed skin. Int J Mol Sci.

[bib9] Chen M., Shamim M.A., Shahid A., Yeung S., Andresen B.T., Wang J. (2020). Topical delivery of carvedilol loaded nano-transfersomes for skin cancer chemoprevention. Pharmaceutics.

[bib10] Chida Y., Hamer M., Wardle J., Steptoe A. (2008). Do stress-related psychosocial factors contribute to cancer incidence and survival?. Nat Clin Pract Oncol.

[bib11] Cleveland K.H., Yeung S., Huang K.M., Liang S., Andresen B.T., Huang Y. (2018). Phosphoproteome profiling provides insight into the mechanism of action for carvedilol-mediated cancer prevention. Mol Carcinog.

[bib12] Congues F., Wang P., Lee J., Lin D., Shahid A., Xie J. (2024). Targeting aryl hydrocarbon receptor to prevent cancer in barrier organs. Biochem Pharmacol.

[bib13] Dhabhar F.S., Saul A.N., Holmes T.H., Daugherty C., Neri E., Tillie J.M. (2012). High-anxious individuals show increased chronic stress burden, decreased protective immunity, and increased cancer progression in a mouse model of squamous cell carcinoma. PLoS One.

[bib14] Elmets C.A., Cala C.M., Xu H. (2014). Photoimmunology. Dermatol Clin.

[bib15] Euvrard S., Kanitakis J., Claudy A. (2003). Skin cancers after organ transplantation. N Engl J Med.

[bib16] Gillbro J.M., Marles L.K., Hibberts N.A., Schallreuter K.U. (2004). Autocrine catecholamine biosynthesis and the beta-adrenoceptor signal promote pigmentation in human epidermal melanocytes. J Invest Dermatol.

[bib17] Gillis R.D., Botteri E., Chang A., Ziegler A.I., Chung N.C., Pon C.K. (2021). Carvedilol blocks neural regulation of breast cancer progression in vivo and is associated with reduced breast cancer mortality in patients. Eur J Cancer.

[bib18] González Maglio D.H., Paz M.L., Leoni J. (2016). Sunlight effects on immune system: is there something else in addition to UV-Induced immunosuppression?. BioMed Res Int.

[bib19] Guereschi M.G., Araujo L.P., Maricato J.T., Takenaka M.C., Nascimento V.M., Vivanco B.C. (2013). Beta2-adrenergic receptor signaling in CD4+ Foxp3+ regulatory T cells enhances their suppressive function in a PKA-dependent manner. Eur J Immunol.

[bib20] Gupta S., Mukhtar H. (2002). Chemoprevention of skin cancer: current status and future prospects. Cancer Metastasis Rev.

[bib21] Hara M.R., Kovacs J.J., Whalen E.J., Rajagopal S., Strachan R.T., Grant W. (2011). A stress response pathway regulates DNA damage through β2-adrenoreceptors and β-arrestin-1. Nature.

[bib22] Honda T., Egen J.G., Lämmermann T., Kastenmüller W., Torabi-Parizi P., Germain R.N. (2014). Tuning of antigen sensitivity by T cell receptor-dependent negative feedback controls T cell effector function in inflamed tissues. Immunity.

[bib23] Huang K.M., Liang S., Yeung S., Oiyemhonlan E., Cleveland K.H., Parsa C. (2017). Topically applied carvedilol attenuates solar ultraviolet radiation induced skin carcinogenesis. Cancer Prev Res (Phila).

[bib24] Hung M., Beazer I.R., Su S., Bounsanga J., Hon E.S., Lipsky M.S. (2022). An exploration of the use and impact of preventive measures on skin cancer. Healthcare (Basel).

[bib25] Liang S., Shamim M.A., Shahid A., Chen M., Cleveland K.H., Parsa C. (2021). Prevention of skin carcinogenesis by the non-β-blocking R-carvedilol enantiomer. Cancer Prev Res (Phila).

[bib26] Lin C.S., Lin W.S., Lin C.L., Kao C.H. (2015). Carvedilol use is associated with reduced cancer risk: a nationwide population-based cohort study. Int J Cardiol.

[bib27] Ma Z., Liu X., Zhang Q., Yu Z., Gao D. (2019). Carvedilol suppresses malignant proliferation of mammary epithelial cells through inhibition of the ROS-mediated PI3K/AKT signaling pathway. Oncol Rep.

[bib28] Miyauchi H., Horio T. (1995). Ultraviolet B-induced local immunosuppression of contact hypersensitivity is modulated by ultraviolet irradiation and hapten application. J Invest Dermatol.

[bib29] Mohammadpour H., MacDonald C.R., Qiao G., Chen M., Dong B., Hylander B.L. (2019). β2 adrenergic receptor-mediated signaling regulates the immunosuppressive potential of myeloid-derived suppressor cells. J Clin Invest.

[bib30] Navid F., Bruhs A., Schuller W., Fritsche E., Krutmann J., Schwarz T. (2013). The aryl hydrocarbon receptor is involved in UVR-induced immunosuppression. J Invest Dermatol.

[bib31] Planta M.B. (2011). Sunscreen and melanoma: is our prevention message correct?. J Am Board Fam Med.

[bib32] Qiao G., Bucsek M.J., Winder N.M., Chen M., Giridharan T., Olejniczak S.H. (2019). β-adrenergic signaling blocks murine CD8^+^ T-cell metabolic reprogramming during activation: a mechanism for immunosuppression by adrenergic stress. Cancer Immunol Immunother.

[bib33] Qiao G., Chen M., Mohammadpour H., MacDonald C.R., Bucsek M.J., Hylander B.L. (2021). Chronic adrenergic stress contributes to metabolic dysfunction and an exhausted phenotype in T cells in the tumor microenvironment. Cancer Immunol Res.

[bib34] Reeve V.E., Allanson M., Domanski D., Painter N. (2012). Gender differences in UV-induced inflammation and immunosuppression in mice reveal male unresponsiveness to UVA radiation. Photochem Photobiol Sci.

[bib35] Ricotti C., Bouzari N., Agadi A., Cockerell C.J. (2009). Malignant skin neoplasms. Med Clin North Am.

[bib36] Ring S., Schäfer S.C., Mahnke K., Lehr H.A., Enk A.H. (2006). CD4+ CD25+ regulatory T cells suppress contact hypersensitivity reactions by blocking influx of effector T cells into inflamed tissue. Eur J Immunol.

[bib37] Rohrer D.K., Chruscinski A., Schauble E.H., Bernstein D., Kobilka B.K. (1999). Cardiovascular and metabolic alterations in mice lacking both beta1- and beta2-adrenergic receptors. J Biol Chem.

[bib38] Sahu R.P., Yao Y., Konger R.L., Travers J.B. (2012). Platelet-activating factor does not mediate UVB-induced local immune suppression. Photochem Photobiol.

[bib39] Saul A.N., Oberyszyn T.M., Daugherty C., Kusewitt D., Jones S., Jewell S. (2005). Chronic stress and susceptibility to skin cancer. J Natl Cancer Inst.

[bib40] Schwarz A., Philippsen R., Schwarz T. (2023). Mouse models of allergic contact dermatitis: practical aspects. J Invest Dermatol.

[bib41] Schwarz T. (2005). Mechanisms of UV-induced immunosuppression. Keio J Med.

[bib42] Schwarz T. (2010). The dark and the sunny sides of UVR-induced immunosuppression: photoimmunology revisited. J Invest Dermatol.

[bib43] Seiffert K., Hosoi J., Torii H., Ozawa H., Ding W., Campton K. (2002). Catecholamines inhibit the antigen-presenting capability of epidermal Langerhans cells. J Immunol.

[bib44] Shahid A., Chen M., Lin C., Andresen B.T., Parsa C., Orlando R. (2023). The β-blocker carvedilol prevents benzo(a)pyrene-induced lung toxicity, inflammation and carcinogenesis. Cancers (Basel).

[bib45] Singh T.P., Vieyra-Garcia P.A., Wagner K., Penninger J., Wolf P. (2018). Cbl-b deficiency provides protection against UVB-induced skin damage by modulating inflammatory gene signature. Cell Death Dis.

[bib46] Sood A.K., Bhatty R., Kamat A.A., Landen C.N., Han L., Thaker P.H. (2006). Stress hormone-mediated invasion of ovarian cancer cells. Clin Cancer Res.

[bib47] Spiegel D., Butler L.D., Giese-Davis J., Koopman C., Miller E., DiMiceli S. (2007). Effects of supportive-expressive group therapy on survival of patients with metastatic breast cancer: a randomized prospective trial. Cancer.

[bib48] Tongkao-On W., Yang C., McCarthy B.Y., De Silva W.G.M., Rybchyn M.S., Gordon-Thomson C. (2021). Sex differences in photoprotective responses to 1,25-dihydroxyvitamin D3 in mice are modulated by the estrogen receptor-β. Int J Mol Sci.

[bib49] Vocanson M., Rozieres A., Hennino A., Poyet G., Gaillard V., Renaudineau S. (2010). Inducible costimulator (ICOS) is a marker for highly suppressive antigen-specific T cells sharing features of TH17/TH1 and regulatory T cells. J Allergy Clin Immunol.

[bib50] Yang E.V., Bane C.M., MacCallum R.C., Kiecolt-Glaser J.K., Malarkey W.B., Glaser R. (2002). Stress-related modulation of matrix metalloproteinase expression. J Neuroimmunol.

[bib51] Yang E.V., Eubank T.D. (2013). The impact of adrenergic signaling in skin cancer progression: possible repurposing of β-blockers for treatment of skin cancer. Cancer Biomark.

[bib52] Yue T.L., Cheng H.Y., Lysko P.G., McKenna P.J., Feuerstein R., Gu J.L. (1992). Carvedilol, a new vasodilator and beta adrenoceptor antagonist, is an antioxidant and free radical scavenger. J Pharmacol Exp Ther.

[bib53] Yue T.L., McKenna P.J., Ruffolo R.R., Feuerstein G. (1992). Carvedilol, a new beta-adrenoceptor antagonist and vasodilator antihypertensive drug, inhibits superoxide release from human neutrophils. Eur J Pharmacol.

